# Author Correction: The high-dimensional space of human diseases built from diagnosis records and mapped to genetic loci

**DOI:** 10.1038/s43588-023-00488-1

**Published:** 2023-06-28

**Authors:** Gengjie Jia, Yu Li, Xue Zhong, Kanix Wang, Milton Pividori, Rabab Alomairy, Aniello Esposito, Hatem Ltaief, Chikashi Terao, Masato Akiyama, Koichi Matsuda, David E. Keyes, Hae Kyung Im, Takashi Gojobori, Yoichiro Kamatani, Michiaki Kubo, Nancy J. Cox, James Evans, Xin Gao, Andrey Rzhetsky

**Affiliations:** 1 Shenzhen Branch, Guangdong Laboratory of Lingnan Modern Agriculture, Genome Analysis Laboratory of the Ministry of Agriculture and Rural Affairs, Agricultural Genomics Institute at Shenzhen, Chinese Academy of Agricultural Sciences, Shenzhen, China; 2 Computational Bioscience Research Center, King Abdullah University of Science and Technology, Thuwal, Saudi Arabia; 3 Computer Science Program, Computer, Electrical and Mathematical Sciences and Engineering Division, King Abdullah University of Science and Technology, Thuwal, Saudi Arabia; 4 Department of Computer Science and Engineering, The Chinese University of Hong Kong, Hong Kong, People’s Republic of China; 5 Department of Medicine and Vanderbilt Genetics Institute, Vanderbilt University Medical Center, Nashville, TN US; 6 Department of Medicine, Institute of Genomics and Systems Biology, Committee on Genomics, Genetics, and Systems Biology, University of Chicago, Chicago, IL US; 7 Department of Operations, Business Analytics, and Information Systems, University of Cincinnati, Cincinnati, OH US; 8 Department of Systems Pharmacology and Translational Therapeutics, Perelman School of Medicine, University of Pennsylvania, Philadelphia, PA US; 9 Extreme Computing Research Center, Computer, Electrical and Mathematical Sciences and Engineering Division, King Abdullah University of Science and Technology, Thuwal, Saudi Arabia; 10 College of Computer Science and Engineering, University of Jeddah, Jeddah, Saudi Arabia; 11 HPE HPC/AI EMEA Research Laboratory, Bristol, UK; 12 RIKEN Center for Integrative Medical Sciences, Yokohama, Japan; 13 Clinical Research Center, Shizuoka General Hospital, Shizuoka, Japan; 14 Department of Applied Genetics, The School of Pharmaceutical Sciences, University of Shizuoka, Shizuoka, Japan; 15 Department of Ophthalmology, Graduate School of Medical Sciences, Kyushu University, Fukuoka, Japan; 16 Department of Computational Biology and Medical Sciences, Graduate School of Frontier Sciences, The University of Tokyo, Tokyo, Japan; 17 Biological and Environmental Science and Engineering Division, King Abdullah University of Science and Technology, Thuwal, Saudi Arabia; 18 Department of Sociology, University of Chicago, Chicago, IL US; 19 Department of Human Genetics, University of Chicago, Chicago, IL US

**Keywords:** Predictive medicine, Genome informatics, Machine learning

Correction to: *Nature Computational Science* 10.1038/s43588-023-00453-y. Published online 22 May 2023.

In the version of this article originally published, the second affiliation for Rabab Alomairy (College of Computer Science and Engineering, University of Jeddah, Jeddah, Saudi Arabia) was listed incorrectly and is now amended in the HTML and PDF versions of the article.

