# Supplementary material for: The high-dimensional space of human diseases built from diagnosis records and mapped to genetic loci
Source: Nat Comput Sci. 2023 May 22;3(5):403–17. doi: 10.1038/s43588-023-00453-y (PMC10766526; doi:10.1038/s43588-023-00453-y)
Supplement: Supplementary file 1 — Supplementary Figs. 1–14 and Tables 1–5. [file 43588_2023_453_MOESM1_ESM.pdf]

nature computational science

Article https://doi.org/10.1038/s43588-023-00453-y

The high-dimensional space of human diseases built from diagnosis records and mapped to genetic loci

In the format provided by the authors and unedited

TABLE OF CONTENTS:

Supplementary Figures 2
Supplementary Tables 15

Supplementary Figure index

Supplementary Figure 1. The overall workflow of this study. 2
Supplementary Figure 2. Annotation for the 20 embedding dimensions by the most separable disease categories. 3
Supplementary Figure 3. Summary of GWAS and replication results. 4
Supplementary Figure 4. Differential associations between blood count records and individuals' embedding scores across 20 dimensions. 5
Supplementary Figure 5. Differential associations between blood biochemistry measurements and individuals' embedding scores across 20 dimensions. 6
Supplementary Figure 6. Differential associations between urine assay measurements and individuals' embedding scores across 20 dimensions. 7
Supplementary Figure 7. Differential associations between spirometry measurements and individuals' embedding scores across 20 dimensions. 7
Supplementary Figure 8. Differential associations between early life factors and individuals' embedding scores across 20 dimensions. 8
Supplementary Figure 9. Differential associations between anthropometry measurements and individuals' embedding scores across 20 dimensions. 8
Supplementary Figure 10. Differential associations between addiction records and individuals' embedding scores across 20 dimensions. 9
Supplementary Figure 11. Differential associations between diet records and individuals' embedding scores across 20 dimensions. 10
Supplementary Figure 12. Differential associations between physical activity/sleep records and individuals' embedding scores across 20 dimensions. 11
Supplementary Figure 13. Differential associations between local environment measurements and individuals' embedding scores across 20 dimensions. 12
Supplementary Figure 14. High concordances between disease similarities out of the spaces with different dimension numbers. 13

Supplementary Table index

Supplementary Table 1. Performance comparisons among polygenic prediction models. 15
Supplementary Table 2. Performance of polygenic prediction models. 17
Supplementary Table 3. Numbers of SNPs in associations with embedding dimensions. 20
Supplementary Table 4. Summary counts of 547 diseases allocated to ten constellations. 21
Supplementary Table 5. Summary counts of constellation assignments for UK Biobank's white British individuals. 21

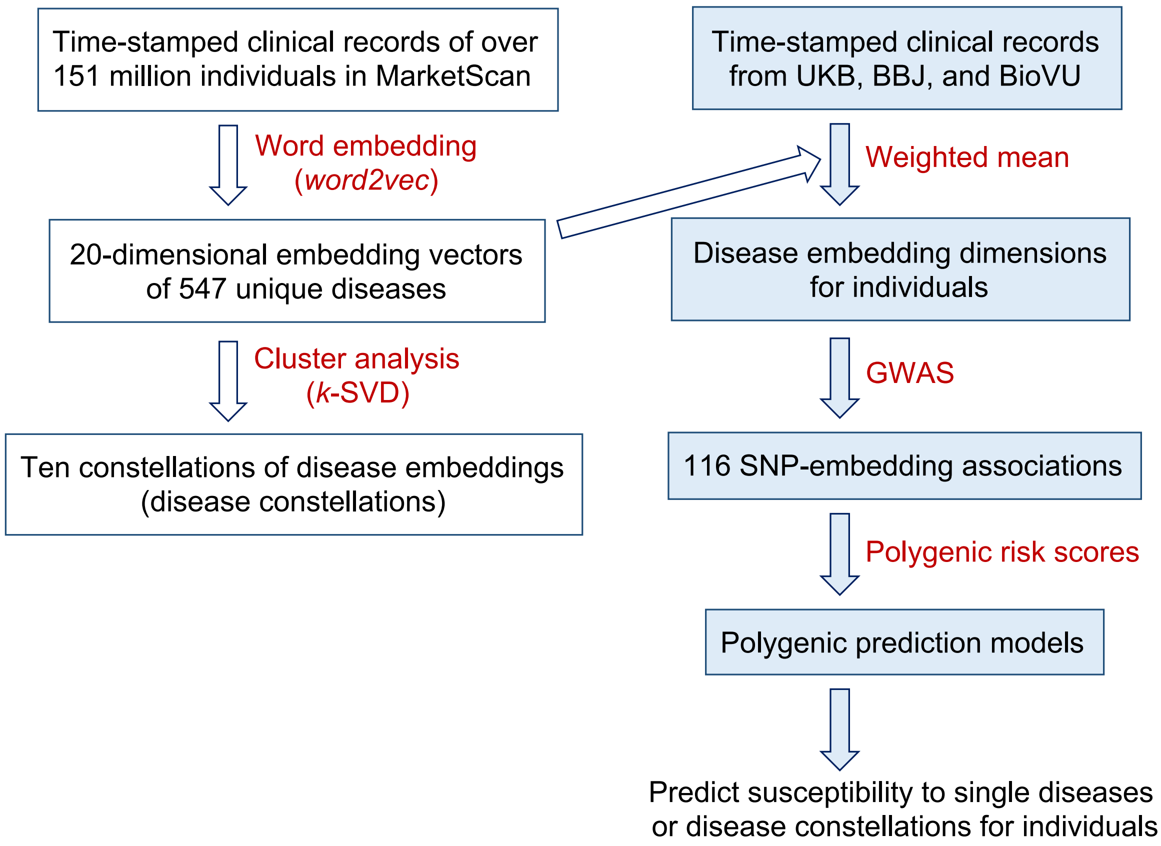

Supplementary Figure 1. The overall workflow of this study.

Our workflow in this study consists of two main streamlines. One is summarized on the left: we started with the time-stamped clinical records of over 151 million individuals in US MarketScan and developed 20-dimensional embedding vectors of 547 unique diseases, leading to a high-resolution representation of the human disease space; furthermore, we made various efforts in interpreting the embedding space, and for example, obtained its thematic understanding by identifying ten constellations of disease embeddings. The other streamline is summarized on the right, aiming to link the disease embeddings to genetics: the inferred embedding space allowed us to describe individual-level health states as space points; through GWAS, we identified 116 SNP-embedding associations; further, the polygenic prediction models built on these associations were predictive of individuals' susceptibility to single diseases as well as disease constellations.

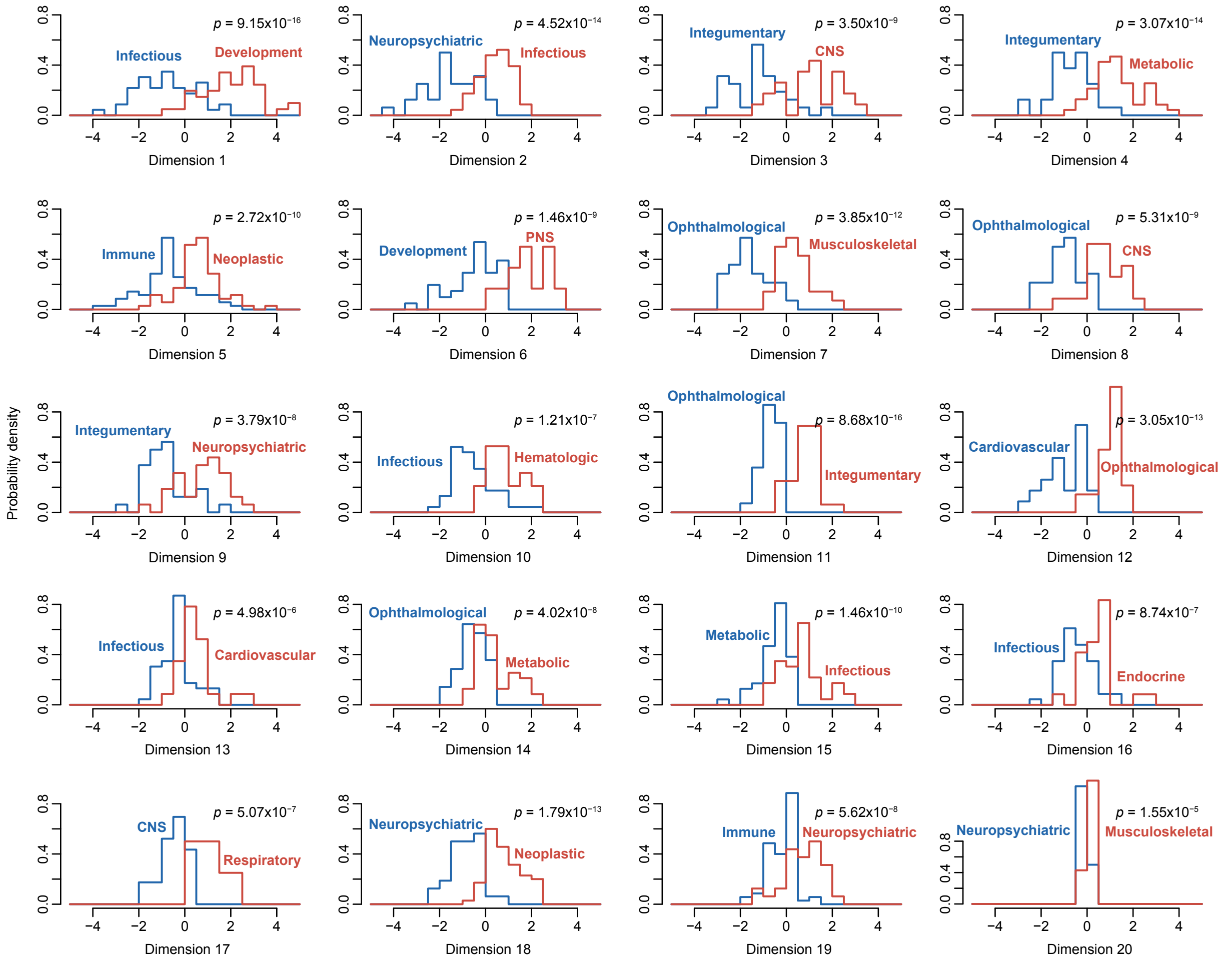

Supplementary Figure 2. Annotation for the 20 embedding dimensions by the most separable disease categories.

As the 547 unique diseases can be grouped into 21 disease categories, we tried to annotate each of the 20 embedding dimensions with the pair of disease categories that were shown most separable in such dimension using the Wilcoxon rank sum test (see Methods section 3). Taking the Dimension 1 as an example, we identified infectious diseases and development disorders as the most separable categories. In the most upper left panel, we show the distribution of embedding values in Dimension 1: infectious diseases are colored in blue, and development disorders are colored in red; the *x*-axis denotes the embedding values, and *y*-axis shows the probability density values; in addition, we report the two-sided *p*-value out of the Wilcoxon rank sum test at upper right corner.

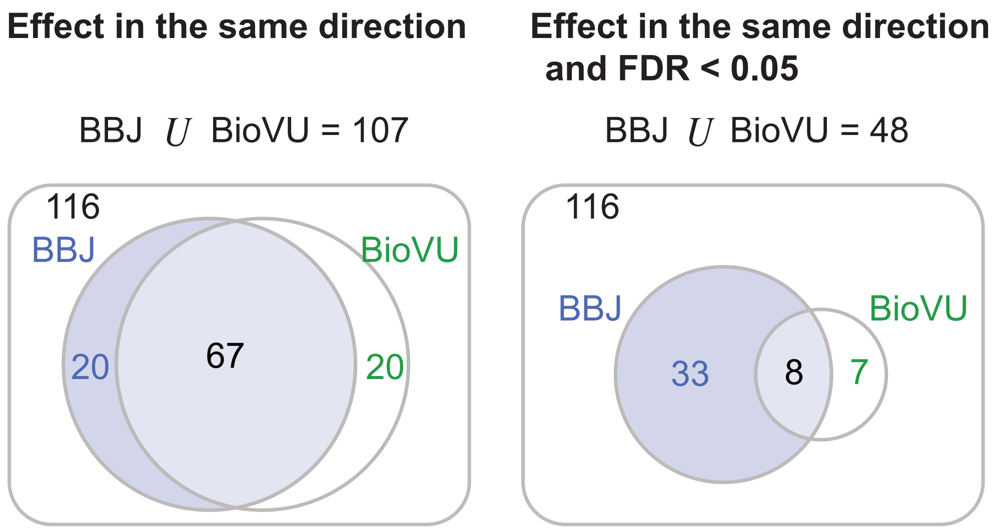

Supplementary Figure 3. Summary of GWAS and replication results.

Using genotype data from the UK Biobank (UKB), we discovered 116 genome-wide significant associations with embedded dimensions, which involve 108 unique loci in total. To replicate these findings, we employed another two cohorts, BioBank Japan (BBJ) and BioVU (from Vanderbilt University in the US). The two Venn diagrams summarize these replication results. First, we checked whether variants had the same effect direction as those seen in the discovery cohort. As shown in the left Venn diagram, the BBJ confirms a set of 87 out of 116 discovered associations, and BioVU confirms another set of 87 associations. The intersection and union of these two sets contain 67 and 107 associations, respectively. Second, we checked whether those associations with consistent effect direction were still significant after controlling the false discovery rate (FDR) via the Benjamini-Hochberg procedure. As a result, there are 41 and 15 associations left in BBJ and in BioVU, respectively (see the Venn diagram on the right), and contains eight common associations. In other words, eight associations are found to be significant in both cohorts, while 48 associations are replicated in at least one cohort.

Blood count

	E1	E2	E3	E4	E5	E6	E7	E8	E9	E10	E11	E12	E13	E14	E15	E16	E17	E18	E19	E20
High light scatter reticulocyte %	−0.82	1.63	5.15	−1.22	5.93	4.34	−4.01	−0.70	3.39	−3.53	−5.26	−2.10	−2.23	1.29	−5.90	7.55	9.95			−0.0190
Red blood cell (erythrocyte) distribution width	−0.68	−0.06	1.01		0.34	0.44	0.19	−0.39	0.25	0.75	−0.13	−0.39	−0.47	−0.17	−0.15	−0.10	0.68	0.13	0.28	
Reticulocyte %	−0.36	0.62	1.30	−0.32	2.14	1.32	−1.47		1.14	−1.18	−1.88	−0.69	−0.73	0.50	−1.93	2.61	3.11			−0.0068
High light scatter reticulocyte count	−0.25	0.48	1.54	−0.37	2.03	1.39	−1.34	−0.19	1.16	−1.37	−1.89	−0.75	−0.81	0.51	−2.00	2.66	3.35		−0.09	−0.0065
Neutrophill count	−0.23	−0.08	1.28	−0.31	0.41	0.21	0.10	−0.25	0.62	−0.45	−0.63	−0.79	−0.27	−0.37	−0.38	0.81	1.99	−0.51	0.37	−0.0029
Mean reticulocyte volume	−0.21		0.59	−0.27	−0.25				0.07	0.34	0.47				−0.08	−0.39	0.20	0.08	0.19	0.0008
Reticulocyte count	−0.17	0.37	0.66	−0.15	1.45	0.79	−0.94		0.82	−0.98	−1.36	−0.47	−0.49	0.40	−1.36	1.85	2.06		−0.20	−0.0048
White blood cell (leukocyte) count		0.40	3.22	−0.94	0.85		−0.36	−0.93	1.86	−1.74	−1.35	−2.01	−0.58	−0.95	−0.92	1.77	4.60	−0.45	0.56	−0.0049
Neutrophill %	−0.15	−0.04	0.18	0.03	0.09	0.09	0.05	−0.05	−0.03	0.11	−0.06	−0.12	−0.08	−0.14	0.08	0.04	0.21	−0.11	0.12	−0.0007
Mean sphered cell volume	−0.12		0.49	−0.38	−0.64	−0.35		0.18		0.32	0.71	0.18	0.33			−0.76	−0.21	0.11	0.33	0.0016
Mean corpuscular hemoglobin		0.22	0.62	−0.81	−0.33	−0.76	−0.72	0.45			0.59		0.68			−0.60		0.24	0.25	0.0017
Mean corpuscular volume	−0.10		0.33	−0.36	−0.16	−0.30	−0.20	0.16		0.10	0.26	−0.07	0.28			−0.32		0.08	0.14	0.0011
Basophill %		−0.15	0.17		−0.25		0.27					−0.17	−0.17			−0.22			0.11	
Basophill count			0.50	−0.13			0.23		0.16	−0.23	−0.28	−0.40	−0.21	−0.14			0.75	−0.15	0.18	
Platelet distribution width	−0.09	0.06	0.09	0.05	0.14	0.09	−0.18		−0.06		−0.06	−0.06	−0.09	−0.08	0.03	0.06	0.10		0.02	−0.0004
Immature reticulocyte fraction	−0.07	0.07	0.48	−0.17	0.43	0.36	−0.23	−0.07	0.29	−0.24	−0.38	−0.18	−0.19	0.13	−0.44	0.54	0.82		0.04	−0.0011
Mean platelet thrombocyte volume		0.03	0.04		0.19	0.09	−0.11		−0.10	−0.04	−0.07	−0.11	−0.07	−0.07	0.05	0.04	0.12	−0.06		−0.0001
Platelet count		−0.28	−0.09		−0.32	−0.08	0.51	−0.16	0.88			0.27	0.21	0.47	−0.61	0.40	0.15		−0.16	
Platelet crit		−0.26	−0.08				0.41	−0.14	0.80	−0.10	−0.18	0.15	0.11	0.42	−0.60	0.52	0.35	−0.07	−0.17	−0.0007
Nucleated red blood cell count																				
Lymphocyte %	0.07	0.12	−0.20	−0.08		−0.11	−0.20	0.21		−0.08	0.09	0.15	0.11	0.24		0.04	−0.38	0.14	−0.15	0.0008
Nucleated red blood cell %										0.34				0.31			−0.27			
Lymphocyte count		1.85	2.48	−1.17	0.78	−0.89	−2.16	−0.71	1.58	−1.98		−1.43		−0.51		1.03	1.81	1.46	−0.30	
Monocyte %	0.13				0.17		−0.28			0.26		−0.13	−0.18		−0.20	−0.29	0.09	0.17		
Hematocrit %	0.24	−0.14	−0.48		−0.12	−0.56	0.21	−0.11	0.30	−0.61	−0.22		0.27	0.25	−0.42	0.16		−0.07	−0.32	
Red blood cell (erythrocyte) count	0.26	−0.13	−0.58	0.22		−0.25	0.29	−0.21	0.24	−0.54	−0.35			0.22	−0.34	0.34		−0.10	−0.35	−0.0006
Hemoglobin concentration	0.27	−0.08	−0.47	−0.05	−0.11	−0.55	0.08		0.28	−0.61	−0.19		0.28	0.28	−0.41	0.18		−0.04	−0.31	
Monocyte count			3.02	−0.92	1.67	0.55	−0.91	−0.47	1.98	−1.06	−2.12	−2.46	−1.19	−1.00	−1.98	1.12	5.31	−0.41	0.58	−0.0071
Mean corpuscular hemoglobin concentration	0.33	0.36	−0.24	−0.16		−0.34	−0.77	0.27		−0.40			0.28	0.29		0.19	−0.25	0.20	−0.15	−0.0014
Eosinophill %	1.92	−0.89	−0.75	0.74	−2.31	−0.16	2.64	−2.24	0.84	−1.73		0.50	0.47	−0.61	−1.30	−0.88	1.27	−0.40	−0.19	0.0025
Eosinophill count	2.25	−1.09		0.64	−2.45		3.17	−2.77	1.48	−2.58	−0.54		0.37	−0.89	−1.87	−0.43	2.86	−0.80		0.0012

β: 2, 1, 0, −1, −2

Supplementary Figure 4. Differential associations between blood count records and individuals' embedding scores across 20 dimensions.

Focusing one of blood count records at a time, we tested its associations with individuals' embedding coordinates along the dimensions from the first (E1) to the 20th (E20) using multivariate regression models (see Methods section 9). This table summarizes the association coefficients, the values of which are color-coded and explicitly written out if they are significantly different from 0. The significance threshold was set as a false discovery rate (FDR) of 0.05.

Blood biochemistry

	E1	E2	E3	E4	E5	E6	E7	E8	E9	E10	E11	E12	E13	E14	E15	E16	E17	E18	E19	E20
Creatinine	−1.24	1.65	2.52	1.24	3.51		−0.71	−1.81		0.91	−0.31	−0.51	−0.73	−0.66	−0.36	1.03	1.78	−0.75	0.52	
Cystatin C	−1.06	0.49	2.80		2.12	0.25	−0.83	−0.68	1.11	0.43	−0.42	−0.51	−0.53		−1.47	1.66	2.43	−0.29	0.27	−0.0008
Alkaline phosphatase	−0.94	−0.48	1.51	−0.80	0.53	−0.26	−0.39		1.88	0.25	−0.71	−0.30	−0.43	0.83	−1.43	1.44	1.15	0.34	−0.15	
Aspartate aminotransferase	−0.80	0.32	1.54	−0.76	0.52		−1.42	0.51	1.42		−0.99		−0.39	1.16	−2.04	1.44	1.97	0.28		−0.0024
Direct bilirubin	−0.66	0.51	1.48	0.33	1.81	0.42	−0.83	−0.41	−1.29	0.37	−1.20	−1.85	−1.16	−1.37	1.57		1.57	−0.84	0.34	−0.0033
Gamma glutamyltransferase	−0.31	0.09	0.96	−0.24	0.30		−0.47	0.32	0.56		−0.76	−0.27	−0.13	0.22	−0.67	0.56	0.79		0.04	−0.0013
C-reactive protein	−0.22	−0.09	0.59	−0.16	0.09	0.04	0.11	−0.14	0.49					0.10	−0.46	0.33	0.63	0.08	0.03	−0.0003
Urea	−0.22	0.53	0.95	−0.13	1.48	0.27	−0.79	−0.36	0.13	−0.14	0.34	0.17		0.11	−0.72	0.95	0.84	0.11		−0.0006
Total bilirubin	−0.14	0.20	−0.12	0.29	0.51	−0.13	−0.15	−0.30	−0.59	0.09	−0.17	−0.40	−0.23	−0.31	0.66	−0.31		−0.14	−0.06	−0.0007
Rheumatoid factor	−0.12	−0.03	0.05	−0.05	−0.07	0.06			0.13	0.05	0.27	0.15		0.05	−0.08		0.17		−0.08	−0.0004
Alanine aminotransferase	−0.12	0.19	0.48	−0.25	0.36	0.27	−0.75		0.76	−0.56	−0.85	0.17	−0.29	0.58	−1.20	1.34	1.11	0.18	−0.24	−0.0027
Testosterone	−0.11	−0.22	−0.17		−0.35	−0.46	0.34			0.33	0.36		0.15	0.10	0.28	−0.38	−0.54	−0.12	−0.07	0.0019
Triglycerides	−0.09	0.04	0.10	−0.10	0.18	0.08	−0.22	0.11	0.32	−0.20	−0.24	0.04		0.32	−0.39	0.45	0.28	0.08	−0.07	−0.0005
Urate	−0.04	0.18	0.25	−0.08	0.53	0.05	−0.26	−0.16	0.34	−0.26	−0.29	−0.05		0.27	−0.68	0.42	0.62	0.07	−0.08	−0.0011
SHBG	−0.03	−0.06		0.09	−0.22	−0.11	0.10	0.10	−0.22	0.31	0.22	−0.04		−0.29	0.37	−0.49	−0.30	−0.06	0.12	0.0007
Apolipoprotein B	−0.02	−0.09	−0.28		−0.22	−0.25	0.14	0.04	0.18	0.04	0.16	0.25	0.19	0.29	−0.17	−0.11	−0.34	0.15	−0.11	0.0009
LDL direct		−0.18	−0.55	0.03	−0.48	−0.49	0.33	0.06	0.26	0.12	0.35	0.49	0.37	0.48	−0.24	−0.30	−0.74	0.26	−0.18	0.0018
Lipoprotein A				−0.02	0.02				0.01	−0.01		−0.02		0.02			0.03		−0.01	
Cholesterol		−0.16	−0.58		−0.53	−0.52	0.32	0.06	0.27	0.12	0.38	0.53	0.41	0.48	−0.30	−0.34	−0.80	0.30	−0.16	0.0018
IGF-1		0.12	−0.25	0.29		−0.06	0.08	−0.16	−0.58	0.06	0.19	−0.11		−0.33	0.74	−0.33	−0.46		0.05	−0.0012
Oestradiol				0.31		0.30	0.27	0.36	−0.55				−0.41	−0.45	0.59	−0.70			0.25	
Vitamin D	0.08	0.22	−0.29	−0.05	0.09	−0.08	0.07	0.11	−0.16	0.20	0.40	0.29	0.12	0.13	0.07	−0.16	−0.33	0.20	−0.10	0.0002
Total protein	0.08	0.21		0.13		0.10	−0.18		0.19	−0.06	−0.11	0.07	−0.15	0.08	−0.33	0.18	0.23		−0.04	−0.0023
Apolipoprotein A	0.09	0.02	−0.21		−0.22	−0.17	0.08	−0.04	−0.03	0.02	0.14	0.18	0.14		−0.09	−0.25	−0.33	0.11	0.02	0.0003
Glucose	0.10	0.39	1.47	−0.29	0.76	1.84	−1.44	−0.60	−0.45	−1.09	−1.18	−0.16	−1.19	−1.20	−0.74	2.61	1.59		0.52	−0.0045
Calcium	0.13	0.25		−0.14	0.32		−0.53	0.13	0.45	−0.29	−0.12	0.23		0.32	−0.76	0.66	0.21	0.20	−0.19	−0.0019
Phosphate	0.14	0.07		−0.47			−0.18	0.33	0.12	0.07	0.34	0.09	0.09	0.56	−0.36			0.12		0.0017
HDL cholesterol	0.14		−0.33		−0.43	−0.30	0.19	−0.05	−0.11	0.13	0.34	0.29	0.23	−0.07		−0.54	−0.63	0.15	0.05	0.0010
Albumin	0.20	0.23	−0.29			−0.04	−0.31	0.15	−0.06	−0.17		0.09		0.10	−0.08	0.04	−0.17	0.08	−0.06	−0.0012
Glycated hemoglobin (HbA1c)	0.54	0.86	5.18	−1.66	2.78	5.88	−4.25	−1.69	−0.75	−3.66	−3.86	−1.10	−3.78	−3.42	−2.77	8.59	6.12	−0.27	1.62	−0.0113

β: 2, 1, 0, −1, −2

Supplementary Figure 5. Differential associations between blood biochemistry measurements and individuals' embedding scores across 20 dimensions.

Focusing one of blood biochemistry measurements at a time, we tested its associations with individuals' embedding coordinates along the dimensions from the first (E1) to the 20th (E20) using multivariate regression models (see Methods section 9). This table summarizes the association coefficients, the values of which are color-coded and explicitly written out if they are significantly different from 0. The significance threshold was set as a false discovery rate (FDR) of 0.05.

Urine assays

	E1	E2	E3	E4	E5	E6	E7	E8	E9	E10	E11	E12	E13	E14	E15	E16	E17	E18	E19	E20
Microalbumin in urine	−0.71	0.70	1.51	0.33	1.30	0.37	−0.65	−0.76			−0.34	−0.19	−0.48	−0.95	−0.23	0.90	0.99	−0.27	0.37	
Creatinine enzymatic in urine	−0.08	−0.07	0.18	−0.13	0.15		−0.06		0.16	−0.12	−0.15		0.06	0.18	−0.18	0.24	0.27			
Sodium in urine	−0.02	0.03	0.04	−0.03	0.06	0.03			0.07	−0.10	−0.11			0.10	−0.08	0.20	0.08		−0.03	−0.0003
Potassium in urine		−0.01		−0.04	0.03	0.01	−0.06			−0.05			0.03	0.03	−0.04	0.05	0.04	0.02	0.02	

β: 2, 1, 0, −1, −2

Supplementary Figure 6. Differential associations between urine assay measurements and individuals' embedding scores across 20 dimensions.

Focusing one of urine assay measurements at a time, we tested its associations with individuals' embedding coordinates along the dimensions from the first (E1) to the 20th (E20) using multivariate regression models (see Methods section 9). This table summarizes the association coefficients, the values of which are color-coded and explicitly written out if they are significantly different from 0. The significance threshold was set as a false discovery rate (FDR) of 0.05.

Spirometry

	E1	E2	E3	E4	E5	E6	E7	E8	E9	E10	E11	E12	E13	E14	E15	E16	E17	E18	E19	E20
FEV1	−0.76	0.73	−1.56	0.55	0.23		−0.69	1.07	−1.27	0.59	0.77	0.68		1.18	1.63		−2.61	0.30	−0.43	−0.0013
FEV1 / $FEV1_{PRD}$	−0.75	0.69	−1.36	0.69	0.15	0.10	−0.53	1.10	−1.44	0.53	0.59	0.52		0.76	1.83	−0.23	−2.32	0.15	−0.25	−0.0019
FVC / FVC_{PRD}	−0.61	0.66	−2.25	0.91	−0.86	−0.38	0.58	0.95	−1.77	0.75	0.88	0.84	0.21	0.85	2.35	−1.12	−3.36		−0.20	
FVC	−0.42	0.46	−1.89	0.52	−0.67	−0.46	0.43	0.59	−1.03	0.57	0.84	0.81	0.34	1.05	1.41	−0.69	−2.75	0.20	−0.33	0.0017
$\frac{FEV1 / FVC}{(FEV1 / FVC)_{PRD}}$	−0.35	0.27	−0.08	0.14	0.56	0.28	−0.75	0.48	−0.38	0.10	0.06	0.04	−0.15	0.26	0.42	0.37	−0.37	0.10	−0.12	−0.0020
FEV1 / FVC	−0.33	0.25	−0.08	0.14	0.53	0.27	−0.71	0.46	−0.36	0.10	0.06	0.04	−0.15	0.24	0.40	0.35	−0.35	0.09	−0.11	−0.0019
PEF / PEF_{PRD}		0.37	−0.73	0.42	−0.16		0.31	0.08	−0.76	−0.10	0.28	0.45		0.35	0.68	−0.30	−0.83	0.05	−0.21	
PEF		0.36	−0.77	0.29	−0.09	−0.10	0.21		−0.54	−0.09	0.36	0.54	0.12	0.57	0.40	−0.17	−0.85	0.18	−0.31	0.0006

β: 2, 1, 0, −1, −2

Supplementary Figure 7. Differential associations between spirometry measurements and individuals' embedding scores across 20 dimensions.

Focusing one of spirometry measurements at a time, we tested its associations with individuals' embedding coordinates along the dimensions from the first (E1) to the 20th (E20) using multivariate regression models (see Methods section 9). This table summarizes the association coefficients, the values of which are color-coded and explicitly written out if they are significantly different from 0. The significance threshold was set as a false discovery rate (FDR) of 0.05.

Early life factors

	E1	E2	E3	E4	E5	E6	E7	E8	E9	E10	E11	E12	E13	E14	E15	E16	E17	E18	E19	E20
Birth weight	−0.07		−0.10	0.04	−0.07	−0.06	0.06	0.05	−0.17	0.07	0.05	0.06			0.13	−0.20	−0.19	0.04	0.04	0.0006
Maternal smoking around birth	−0.01	0.01	0.02	−0.02	0.02			0.03	0.03			0.01		0.03	−0.03	0.03	0.03		−0.01	0.0001
Breastfed as a baby		0.01					−0.01		−0.01	−0.01	0.01	0.01	0.01		−0.01		−0.01	0.02		−0.0001

β: 0.1, 0.05, 0, −0.05, −0.1

Supplementary Figure 8. Differential associations between early life factors and individuals' embedding scores across 20 dimensions.

Focusing one of early life factors at a time, we tested its associations with individuals' embedding coordinates along the dimensions from the first (E1) to the 20th (E20) using multivariate regression models (see Methods section 9). This table summarizes the association coefficients, the values of which are color-coded and explicitly written out if they are significantly different from 0. The significance threshold was set as a false discovery rate (FDR) of 0.05.

Anthropometry

	E1	E2	E3	E4	E5	E6	E7	E8	E9	E10	E11	E12	E13	E14	E15	E16	E17	E18	E19	E20
Sitting height	−0.07		−0.27	0.25		−0.06	−0.29	−0.19	−0.70	−0.27		0.08		−0.39	0.34	−0.34	−0.18	0.24	0.17	0.0005
Weight	−0.04	0.13	0.44	−0.07	0.57	0.39	−0.40	0.07	0.11	−0.50	−0.33	0.09		0.30	−0.80	0.84	0.74	0.09	−0.03	−0.0010
Whole body fat mass	−0.04	0.06	0.42	−0.06	0.50	0.35	−0.30	0.08	0.24	−0.45	−0.38	0.03	−0.03	0.29	−0.72	0.82	0.77		−0.05	−0.0010
Waist circumference	−0.04	0.07	0.61	−0.10	0.61	0.47	−0.35	0.04	0.33	−0.56	−0.56		−0.04	0.32	−0.91	1.09	1.04		−0.02	−0.0014
Basal metabolic rate	−0.04	0.22	0.42	−0.08	0.61	0.43	−0.53	0.05	−0.08	−0.54	−0.23	0.19	0.09	0.28	−0.86	0.82	0.62	0.20		−0.0010
BMI	−0.03	0.15	0.55	−0.12	0.66	0.49	−0.41	0.12	0.31	−0.59	−0.45	0.07		0.46	−0.97	1.12	0.96	0.04	−0.09	−0.0015
Hip circumference		0.08	0.60	−0.10	0.76	0.49	−0.48	0.06	0.28	−0.63	−0.40	0.14	0.08	0.46	−1.11	1.15	1.00	0.10	−0.08	−0.0013
Body fat %		0.03	0.31	−0.05	0.40	0.26	−0.21	0.10	0.32	−0.35	−0.35		−0.06	0.28	−0.59	0.68	0.69		−0.08	−0.0009

β: 1, 0.5, 0, −0.5, −1

Supplementary Figure 9. Differential associations between anthropometry measurements and individuals' embedding scores across 20 dimensions.

Focusing one of anthropometry measurements at a time, we tested its associations with individuals' embedding coordinates along the dimensions from the first (E1) to the 20th (E20) using multivariate regression models (see Methods section 9). This table summarizes the association coefficients, the values of which are color-coded and explicitly written out if they are significantly different from 0. The significance threshold was set as a false discovery rate (FDR) of 0.05.

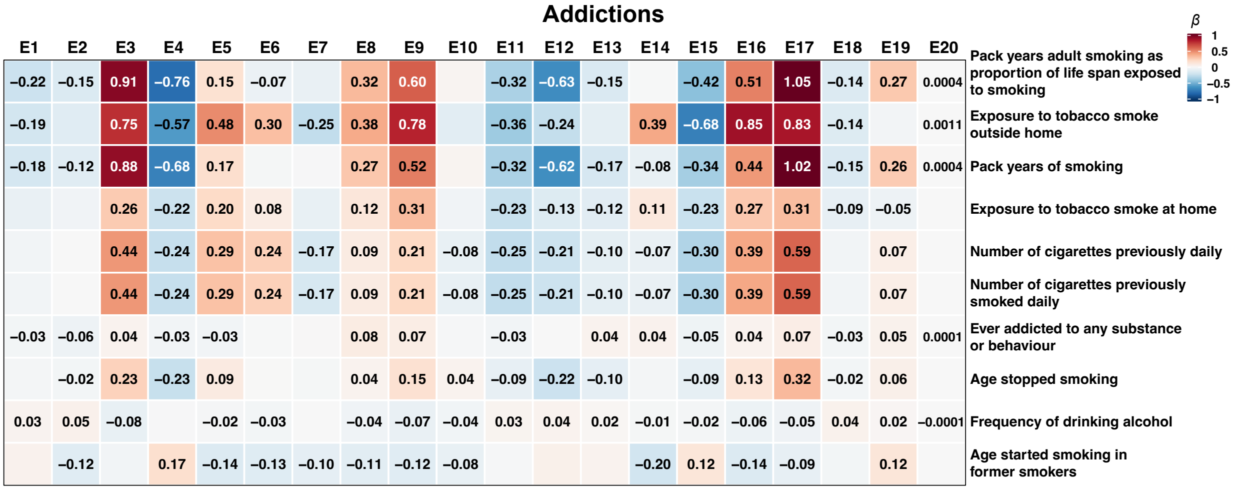

	E1	E2	E3	E4	E5	E6	E7	E8	E9	E10	E11	E12	E13	E14	E15	E16	E17	E18	E19	E20
Pack years adult smoking as proportion of life span exposed to smoking	−0.22	−0.15	0.91	−0.76	0.15	−0.07		0.32	0.60		−0.32	−0.63	−0.15		−0.42	0.51	1.05	−0.14	0.27	0.0004
Exposure to tobacco smoke outside home	−0.19		0.75	−0.57	0.48	0.30	−0.25	0.38	0.78		−0.36	−0.24		0.39	−0.68	0.85	0.83	−0.14		0.0011
Pack years of smoking	−0.18	−0.12	0.88	−0.68	0.17			0.27	0.52		−0.32	−0.62	−0.17	−0.08	−0.34	0.44	1.02	−0.15	0.26	0.0004
Exposure to tobacco smoke at home			0.26	−0.22	0.20	0.08		0.12	0.31		−0.23	−0.13	−0.12	0.11	−0.23	0.27	0.31	−0.09	−0.05	
Number of cigarettes previously daily			0.44	−0.24	0.29	0.24	−0.17	0.09	0.21	−0.08	−0.25	−0.21	−0.10	−0.07	−0.30	0.39	0.59		0.07	
Number of cigarettes previously smoked daily			0.44	−0.24	0.29	0.24	−0.17	0.09	0.21	−0.08	−0.25	−0.21	−0.10	−0.07	−0.30	0.39	0.59		0.07	
Ever addicted to any substance or behaviour	−0.03	−0.06	0.04	−0.03	−0.03			0.08	0.07		−0.03		0.04	0.04	−0.05	0.04	0.07	−0.03	0.05	0.0001
Age stopped smoking		−0.02	0.23	−0.23	0.09			0.04	0.15	0.04	−0.09	−0.22	−0.10		−0.09	0.13	0.32	−0.02	0.06	
Frequency of drinking alcohol	0.03	0.05	−0.08		−0.02	−0.03		−0.04	−0.07	−0.04	0.03	0.04	0.02	−0.01	−0.02	−0.06	−0.05	0.04	0.02	−0.0001
Age started smoking in former smokers		−0.12		0.17	−0.14	−0.13	−0.10	−0.11	−0.12	−0.08				−0.20	0.12	−0.14	−0.09		0.12	

Supplementary Figure 10. Differential associations between addiction records and individuals' embedding scores across 20 dimensions.

Focusing one of addiction records at a time, we tested its associations with individuals' embedding coordinates along the dimensions from the first (E1) to the 20th (E20) using multivariate regression models (see Methods section 9). This table summarizes the association coefficients, the values of which are color-coded and explicitly written out if they are significantly different from 0. The significance threshold was set as a false discovery rate (FDR) of 0.05.

Diet

E1	E2	E3	E4	E5	E6	E7	E8	E9	E10	E11	E12	E13	E14	E15	E16	E17	E18	E19	E20	
−0.10		0.09	−0.11	0.09	0.16		−0.12	0.13		−0.25	−0.20	−0.11	−0.07	−0.10	0.31	0.20	−0.10	0.06		Bread intake
−0.10	−0.11	0.08	−0.07			0.22	0.33	0.29	0.24				0.23				−0.07		0.0014	Tea intake
−0.03	−0.02	0.02	−0.05	−0.03	−0.02	0.06	0.05	0.05	0.04		0.02	0.02	0.07	−0.01			0.01		0.0004	Salt added to food
−0.02	0.05	0.03	−0.03	0.05					−0.02	−0.04		−0.03			0.05	0.03	0.03	−0.01	−0.0001	Beef intake
		0.20		0.24	0.06	−0.17	−0.14	0.20	−0.14		0.14	0.13	0.12	−0.35	0.13	0.17	0.08		0.0006	Water intake
	0.04	−0.03	−0.01	0.06					−0.04	−0.03	0.02		0.08	−0.03	0.06	0.04	0.02	−0.04		Poultry intake
		0.03	−0.02		0.01		0.01	0.03	−0.03		0.01	0.01	0.04	−0.05	0.05	0.05				Variation in diet
	0.03		−0.03	0.06		−0.03								−0.02	0.02	0.03	0.02		−0.0001	Non-oily fish intake
	0.01	0.03			0.02	0.02	−0.02	0.02	−0.03	−0.05	−0.01		0.01	−0.04	0.06	0.05	−0.01	0.02	−0.0001	Processed meat intake
	0.04	0.05	−0.05	0.04		−0.03		0.03	−0.04	−0.04			0.04	−0.10	0.10	0.06	0.03	−0.01		Pork intake
	0.03	0.04	−0.03	0.03	−0.02	−0.03	−0.05	0.02	−0.04	−0.03		0.02		−0.09	0.05	0.04	0.05			Lamb or mutton intake
	0.16		−0.08	0.12		−0.15	0.08	−0.09	−0.12	0.24	0.19	0.12	0.15	−0.18		−0.08	0.14			Salad or raw vegetable intake
0.02	0.03	−0.02	−0.03	0.04		−0.05	−0.02	−0.03	−0.02	0.02			−0.02	−0.02	−0.03	0.02	0.02		−0.0001	Oily fish intake
0.02	−0.02	−0.05	0.06	−0.10	−0.04	0.05	−0.07	−0.10	0.01	0.03	0.02	0.02	−0.09	0.05	−0.11	−0.09		0.05		Cheese intake
0.06	−0.09	−0.29	0.19		0.10		−0.10	−0.25	0.15	0.14			−0.19	0.31	−0.13	−0.37		−0.08	0.0008	Cereal intake
0.11	0.08	0.11	−0.08	0.07	0.11	−0.10		−0.12	−0.06	0.13	0.10	0.11		−0.11	0.08		0.07			Cooked vegetable intake
0.16		0.23	−0.36	−0.28		−0.19	0.23		−0.17	0.21	0.07	0.08		−0.45	0.23	0.20	0.11	0.07	−0.001	Coffee intake
0.17	0.14	−0.13	−0.09	0.21	0.28	−0.43		−0.11	−0.15	0.24	0.30			−0.34	0.20		0.19	−0.11		Fresh fruit intake
0.19	−0.15	−0.41		−0.26	−0.21		−0.21	−0.15	0.19	0.55	0.23	0.37		0.18	−0.48	−0.72	0.15		0.0014	Dried fruit intake

β: 0.4, 0.2, 0, −0.2, −0.4

Supplementary Figure 11. Differential associations between diet records and individuals' embedding scores across 20 dimensions.

Focusing one of diet records at a time, we tested its associations with individuals' embedding coordinates along the dimensions from the first (E1) to the 20th (E20) using multivariate regression models (see Methods section 9). This table summarizes the association coefficients, the values of which are color-coded and explicitly written out if they are significantly different from 0. The significance threshold was set as a false discovery rate (FDR) of 0.05.

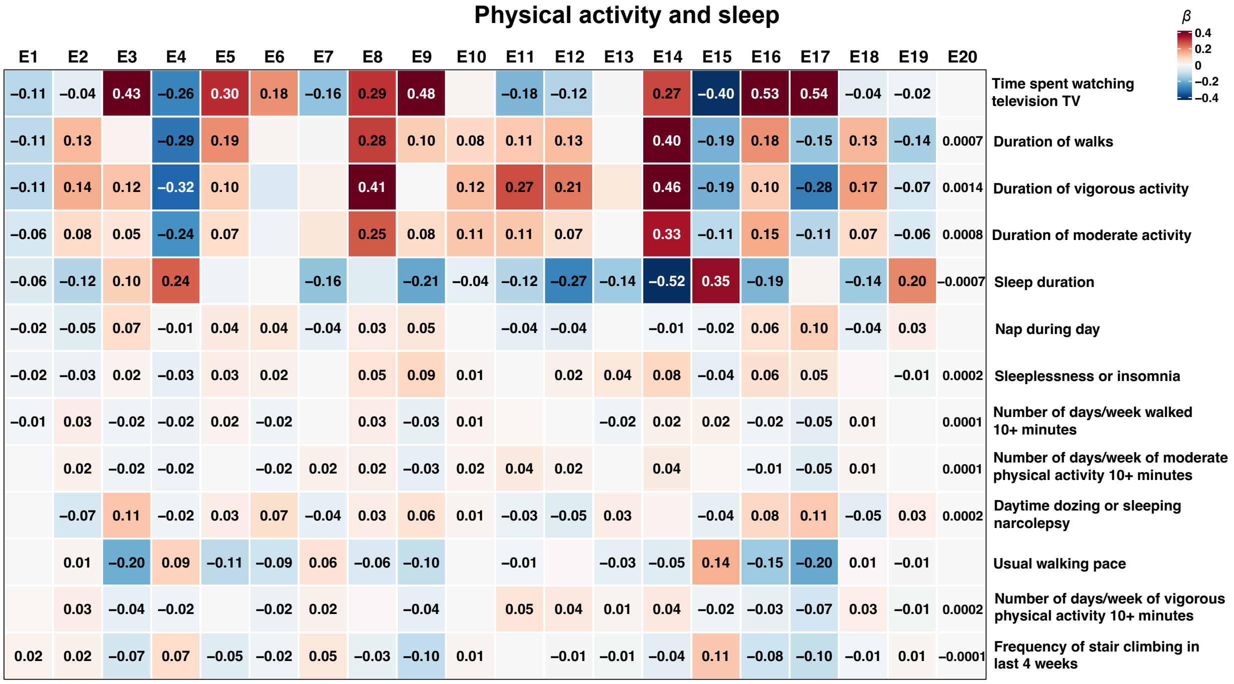

Supplementary Figure 12. Differential associations between physical activity/sleep records and individuals' embedding scores across 20 dimensions.

Focusing one of physical activity/sleep records at a time, we tested its associations with individuals' embedding coordinates along the dimensions from the first (E1) to the 20th (E20) using multivariate regression models (see Methods section 9). This table summarizes the association coefficients, the values of which are color-coded and explicitly written out if they are significantly different from 0. The significance threshold was set as a false discovery rate (FDR) of 0.05.

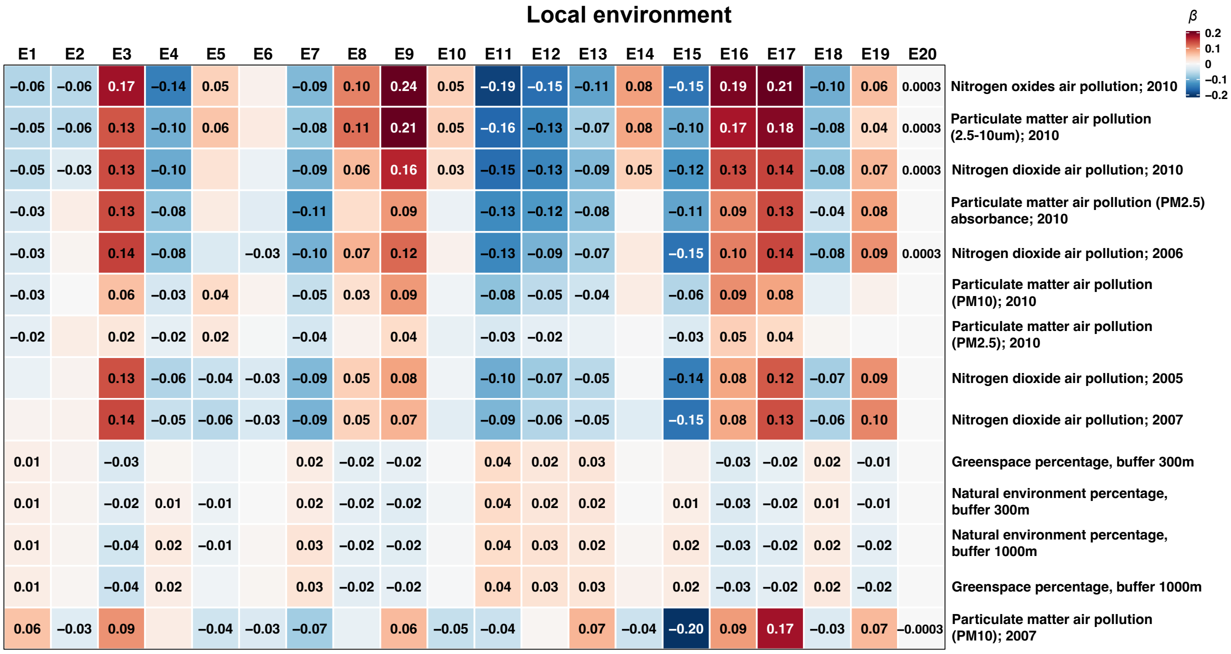

Supplementary Figure 13. Differential associations between local environment measurements and individuals' embedding scores across 20 dimensions.

Focusing one of local environment measurements at a time, we tested its associations with individuals' embedding coordinates along the dimensions from the first (E1) to the 20th (E20) using multivariate regression models (see Methods section 9). This table summarizes the association coefficients, the values of which are color-coded and explicitly written out if they are significantly different from 0. The significance threshold was set as a false discovery rate (FDR) of 0.05.

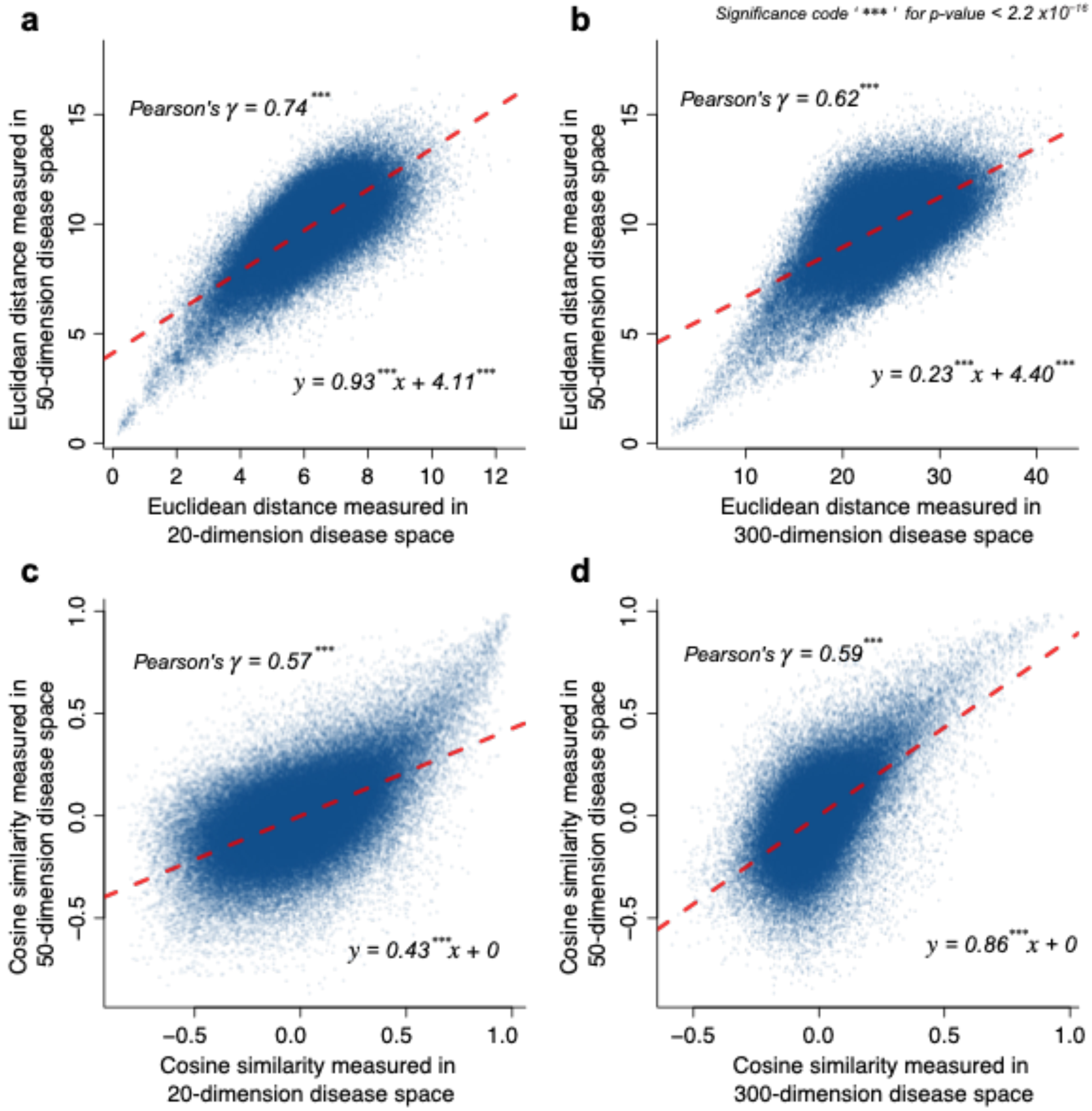

Supplementary Figure 14. High concordances between disease similarities out of the spaces with different dimension numbers.

Regarding the choice of space dimension numbers, in addition to the 20 dimensions currently used, we also tried different dimension numbers, such as 50 and 300. **(a) Euclidean distances measured in 20- and 50-dimension disease spaces:** The disease embeddings were expressed as 20- or 50-dimension vectors, and we computed the Euclidean distances between any two diseases of over 547 diseases, which were measured in 20-dimension space (as plotted along *x*-axis) and in 50-dimension space (as plotted along *y*-axis). To check their concordance, we computed Pearson's correlation coefficient *γ*, and obtained a linear approximation model (shown as a red dashed line. In a similar way, we also measured Euclidean distances in a 300-dimension disease space and compared them with those measured in 50-dimension

space, as shown in **(b)**. Instead of Euclidean distances, we also tried cosine similarities for these comparison purposes, shown in **(c)** and **(d)**. For the correlation coefficients, the linear regression coefficients and the intercepts, we applied the Student's *t* tests to determine their significance levels for being different from 0. We found all of them were very significant with two-sided *p*-values $< 2.2\times10^{-16}$, indicated by three superscript asterisks.

Supplementary Table 1. Performance comparisons among polygenic prediction models.

We compare three predication models (as described in Methods section 10): a conventional Generalized Linear Model (GLM) built on disease-specific GWAS Catalog loci, and two embedding models built on the 116 SNP associations identified in this study, including a Generalized Linear Model (GLM) and a Gradient Boosting Model (GBM). As demonstrations, we show the 95 percent confidence intervals of model performance indices for five common diseases that are among the most prevalent diseases in the general European population and UK Biobank cohort (see the first-to-sixth columns). The counts of respective cases in UK Biobank are shown in parentheses in the first column; Nagelkerke R^2 values from historical analyses was available for three diseases and can serve as baseline values, shown in the third column. The results for many other prevalent diseases can be found in Supplementary Table 2. In addition, given the 20-dimensional embedding vector of the disease shown here, we computed its cosine similarity with respect to each of the ten disease constellations and then claimed that the disease would belong to the constellation with which it has the largest cosine similarity value (see the serial number in the seventh column). In this regard, we predicted whether an individual would likely carry any diseases that belong to the disease constellation and report the performance indices of our embedding models in the eighth and ninth columns here as well as in Table 2.

**Disease** (case count)	**Performance index**	**Published baseline**	**GWAS Catalog: GLM**	**Embedding model: GLM**	**Embedding model: GBM**	**Disease constellation**	**Embedding model: GLM**	**Embedding model: GBM**
**Asthma** (46,952)	Nagelkerke R^2 (*p*-value)	0.025 [d]	0.009 ± 0.001 (0.035)	0.028 ± 0.001 (7.5×10^{-12})	0.057 ± 0.001 (< 10^{-16})	3	0.037 ± 0.001 (< 10^{-16})	0.064 ± 0.001 (< 10^{-16})
	Prediction accuracy [a]	–	51.3% ± 0.1%	54.2% ± 0.1%	54.3% ± 0.2%		55.4% ± 0.1%	55.6% ± 0.1%
	PPV [b]	–	51.3% ± 0.1%	54.2% ± 0.1%	54.4% ± 0.2%		55.3% ± 0.1%	56.1% ± 0.1%
	NPV [c]	–	51.3% ± 0.1%	54.2% ± 0.1%	54.2% ± 0.2%		55.5% ± 0.1%	55.3% ± 0.1%
**Allergic rhinitis** (21,085)	Nagelkerke R^2 (*p*-value)	–	0.045 ± 0.001 (< 10^{-16})	0.050 ± 0.001 (< 10^{-16})	0.075 ± 0.001 (< 10^{-16})	3	0.037 ± 0.001 (< 10^{-16})	0.064 ± 0.001 (< 10^{-16})
	Prediction accuracy	–	56.9% ± 0.1%	56.8% ± 0.1%	57.0% ± 0.1%		55.4% ± 0.1%	55.6% ± 0.1%
	PPV	–	57.1% ± 0.1%	56.9% ± 0.1%	57.0% ± 0.1%		55.3% ± 0.1%	56.1% ± 0.1%
	NPV	–	56.8% ± 0.1%	56.6% ± 0.1%	57.0% ± 0.1%		55.5% ± 0.1%	55.3% ± 0.1%
**Depression** (25,782)	Nagelkerke R^2 (*p*-value)	0.010 ~ 0.011 [e]	0.036 ± 0.001 (< 10^{-16})	0.039 ± 0.001 (< 10^{-16})	0.067 ± 0.001 (< 10^{-16})	10	0.074 ± 0.001 (< 10^{-16})	0.098 ± 0.001 (< 10^{-16})

	Prediction accuracy	–	56.0% ± 0.2%	55.8% ± 0.1%	56.1% ± 0.2%		59.4% ± 0.1%	59.7% ± 0.1%
	PPV	–	55.8% ± 0.1%	55.6% ± 0.1%	56.4% ± 0.2%		59.2% ± 0.1%	59.0% ± 0.1%
	NPV	–	56.1% ± 0.2%	55.9% ± 0.1%	55.9% ± 0.2%		59.5% ± 0.1%	60.6% ± 0.1%
**General hypertensio n** (106,674)	Nagelkerke R^2 (*p*-value)	0.035 [f]	0.117 ± 0.001 (< 10^{-16})	0.123 ± 0.001 (< 10^{-16})	0.140 ± 0.001 (< 10^{-16})	6	0.160 ± 0.001 (< 10^{-16})	0.176 ± 0.001 (< 10^{-16})
	Prediction accuracy	–	61.9% ± 0.1%	61.9% ± 0.1%	62.1% ± 0.1%		64.4% ± 0.1%	64.5% ± 0.1%
	PPV	–	61.1% ± 0.1%	61.2% ± 0.1%	60.7% ± 0.1%		63.6% ± 0.1%	63.9% ± 0.2%
	NPV	–	62.8% ± 0.1%	62.8% ± 0.1%	64.0% ± 0.2%		65.4% ± 0.2%	65.3% ± 0.2%
**Osteoarthrit is** (54,574)	Nagelkerke R^2 (*p*-value)	–	0.102 ± 0.001 (< 10^{-16})	0.105 ± 0.001 (< 10^{-16})	0.127 ± 0.001 (< 10^{-16})	6	0.160 ± 0.001 (< 10^{-16})	0.176 ± 0.001 (< 10^{-16})
	Prediction accuracy	–	60.8% ± 0.1%	60.7% ± 0.2%	60.9% ± 0.1%		64.4% ± 0.1%	64.5% ± 0.1%
	PPV	–	59.7% ± 0.1%	59.7% ± 0.1%	58.8% ± 0.1%		63.6% ± 0.1%	63.9% ± 0.2%
	NPV	–	62.1% ± 0.2%	62.0% ± 0.2%	64.1% ± 0.2%		65.4% ± 0.2%	65.3% ± 0.2%

[a] We defined prediction accuracy as the number of correctly-classified samples to the total number of trials, based on the testing datasets;

[b] PPV: Positive predictive value; we defined it as the proportion of positive results (predicted as cases who had the disease of interest by polygenic prediction models) that are true positive, based on the testing datasets;

[c] NPV: Negative predictive value; we defined it as the proportion of negative results (predicted as controls who did not have the disease of interest by polygenic prediction models) that are true negative, based on the testing datasets;

[d] Reference: PMID 29333270; [e] Reference: PMIDs 20567237, 25993607; [f] Reference: PMID 31049317.

Supplementary Table 2. Performance of polygenic prediction models.

After Supplementary Table 1, We continue on showing the 95 percent confidence intervals of model performance indices for other disease that are highly prevalent in the general European population and UK Biobank cohort (the counts of respective cases in UK Biobank are shown in parentheses). Three predication models are involved (as described in Methods section 10): a conventional Generalized Linear Model (GLM) built on disease-specific GWAS Catalog loci, and two embedding models built on the 116 SNP associations identified in this study, including a Generalized Linear Model (GLM) and a Gradient Boosting Model (GBM).

**Disease** (case count)	**Performance index**	**GWAS Catalog: GLM**	**Embedding model: GLM**	**Embedding model: GBM**
**Hernia** (42,733)	Nagelkerke R^2 (*p*-value)	0.086 ± 0.001 (< 10^{-16})	0.089 ± 0.001 (< 10^{-16})	0.109 ± 0.001 (< 10^{-16})
	Prediction accuracy [a]	59.8% ± 0.1%	59.8% ± 0.1%	59.9% ± 0.1%
	PPV [b]	59.9% ± 0.1%	59.8% ± 0.1%	60.0% ± 0.2%
	NPV [c]	59.8% ± 0.1%	59.8% ± 0.1%	59.8% ± 0.2%
**Non-Specified Cardiac Ischemia** (27,000)	Nagelkerke R^2 (*p*-value)	0.207 ± 0.002 (< 10^{-16})	0.218 ± 0.002 (< 10^{-16})	0.226 ± 0.002 (< 10^{-16})
	Prediction accuracy	66.6% ± 0.2%	66.7% ± 0.1%	66.8% ± 0.1%
	PPV	65.7% ± 0.2%	65.9% ± 0.1%	65.4% ± 0.2%
	NPV	67.6% ± 0.2%	67.6% ± 0.2%	68.6% ± 0.2%
**Functional Digestive Disorder** (26,427)	Nagelkerke R^2 (*p*-value)	0.033 ± 0.001 (< 10^{-16})	0.037 ± 0.001 (< 10^{-16})	0.063 ± 0.001 (< 10^{-16})
	Prediction accuracy	55.6% ± 0.1%	55.5% ± 0.1%	55.5% ± 0.1%
	PPV	55.4% ± 0.1%	55.3% ± 0.1%	55.3% ± 0.1%
	NPV	55.8% ± 0.2%	55.7% ± 0.1%	55.8% ± 0.1%
**Cardiac Dysrhythmia** (21,911)	Nagelkerke R^2 (*p*-value)	0.127 ± 0.001 (< 10^{-16})	0.112 ± 0.001 (< 10^{-16})	0.132 ± 0.001 (< 10^{-16})
	Prediction accuracy	62.6% ± 0.1%	61.7% ± 0.1%	62.0% ± 0.1%
	PPV	61.9% ± 0.1%	60.9% ± 0.1%	61.8% ± 0.2%
	NPV	63.4% ± 0.2%	62.6% ± 0.1%	62.1% ± 0.2%
**Cataract** (19,385)	Nagelkerke R^2 (*p*-value)	0.211 ± 0.002 (< 10^{-16})	0.215 ± 0.002 (< 10^{-16})	0.230 ± 0.002 (< 10^{-16})
	Prediction accuracy	66.5% ± 0.1%	66.4% ± 0.1%	66.5% ± 0.1%
	PPV	64.3% ± 0.1%	64.3% ± 0.1%	64.5% ± 0.1%
	NPV	69.5% ± 0.2%	69.3% ± 0.1%	69.2% ± 0.2%
**Substance Abuse** (17,301)	Nagelkerke R^2 (*p*-value)	0.035 ± 0.001 (< 10^{-16})	0.037 ± 0.001 (< 10^{-16})	0.064 ± 0.001 (< 10^{-16})
	Prediction accuracy [f]	55.9% ± 0.1%	55.7% ± 0.1%	55.7% ± 0.1%
	PPV	55.9% ± 0.1%	55.7% ± 0.1%	55.7% ± 0.1%

	NPV	55.9% ± 0.1%	55.7% ± 0.1%	55.8% ± 0.2%
**Biliary Tract Disease** (17,678)	Nagelkerke R^2 (*p*-value)	0.074 ± 0.001 (< 10^{-16})	0.088 ± 0.001 (< 10^{-16})	0.108 ± 0.001 (< 10^{-16})
	Prediction accuracy	59.3% ± 0.1%	59.8% ± 0.1%	60.0% ± 0.1%
	PPV	59.0% ± 0.1%	59.5% ± 0.1%	59.5% ± 0.2%
	NPV	59.6% ± 0.1%	60.2% ± 0.1%	60.6% ± 0.1%
**Type II Diabetes Mellitus** (15,643)	Nagelkerke R^2 (*p*-value)	0.118 ± 0.001 (< 10^{-16})	0.129 ± 0.001 (< 10^{-16})	0.143 ± 0.001 (< 10^{-16})
	Prediction accuracy	62.0% ± 0.1%	62.3% ± 0.1%	62.5% ± 0.1%
	PPV	61.5% ± 0.1%	61.8% ± 0.1%	61.6% ± 0.1%
	NPV	62.6% ± 0.2%	62.8% ± 0.1%	63.6% ± 0.2%
**Non-Specific Anemia** (16,139)	Nagelkerke R^2 (*p*-value)	0.026 ± 0.001 (8.0×10^{-15})	0.031 ± 0.001 (5.6×10^{-15})	0.066 ± 0.001 (< 10^{-16})
	Prediction accuracy	53.8% ± 0.1%	54.2% ± 0.1%	55.5% ± 0.1%
	PPV	53.8% ± 0.1%	54.2% ± 0.1%	54.7% ± 0.1%
	NPV	53.8% ± 0.1%	54.3% ± 0.1%	56.8% ± 0.2%
**Cerebrovascular Disease** (15,297)	Nagelkerke R^2 (*p*-value)	0.081 ± 0.001 (< 10^{-16})	0.084 ± 0.001 (< 10^{-16})	0.106 ± 0.001 (< 10^{-16})
	Prediction accuracy [f]	59.7% ± 0.1%	59.5% ± 0.1%	59.7% ± 0.1%
	PPV	58.8% ± 0.1%	58.8% ± 0.1%	59.4% ± 0.1%
	NPV	60.7% ± 0.1%	60.4% ± 0.1%	60.0% ± 0.2%
**Melanoma** (15,768)	Nagelkerke R^2 (*p*-value)	0.078 ± 0.001 (< 10^{-16})	0.090 ± 0.001 (< 10^{-16})	0.111 ± 0.001 (< 10^{-16})
	Prediction accuracy	59.5% ± 0.1%	59.8% ± 0.1%	60.1% ± 0.1%
	PPV	58.3% ± 0.1%	58.9% ± 0.1%	59.0% ± 0.1%
	NPV	61.0% ± 0.2%	60.9% ± 0.1%	61.5% ± 0.2%
**Acquired Hypothyroidism** (19,894)	Nagelkerke R^2 (*p*-value)	0.164 ± 0.001 (< 10^{-16})	0.186 ± 0.001 (< 10^{-16})	0.197 ± 0.001 (< 10^{-16})
	Prediction accuracy	65.6% ± 0.1%	66.1% ± 0.1%	66.2% ± 0.1%
	PPV	62.6% ± 0.1%	63.7% ± 0.1%	63.4% ± 0.1%
	NPV	70.4% ± 0.2%	69.4% ± 0.2%	70.6% ± 0.2%
**Esophageal Disease** (33,390)	Nagelkerke R^2 (*p*-value)	0.031 ± 0.001 (< 10^{-16})	0.034 ± 0.001 (< 10^{-16})	0.062 ± 0.001 (< 10^{-16})
	Prediction accuracy	55.1% ± 0.1%	54.9% ± 0.1%	55.3% ± 0.1%
	PPV	54.8% ± 0.1%	54.7% ± 0.1%	54.6% ± 0.1%
	NPV	55.4% ± 0.2%	55.2% ± 0.1%	56.1% ± 0.2%
**Diverticulosis Diverticulitis** (23,800)	Nagelkerke R^2 (*p*-value)	0.108 ± 0.001 (< 10^{-16})	0.111 ± 0.001 (< 10^{-16})	0.133 ± 0.001 (< 10^{-16})
	Prediction accuracy	60.9% ± 0.1%	60.8% ± 0.1%	61.1% ± 0.1%

	PPV	59.7% ± 0.1%	59.6% ± 0.1%	58.9% ± 0.1%
	NPV	62.6% ± 0.1%	62.4% ± 0.2%	64.7% ± 0.2%
**Gastritis Duodenitis** (23,580)	Nagelkerke R^2 (*p*-value)	0.047 ± 0.001 (< 10^{-16})	0.050 ± 0.001 (< 10^{-16})	0.074 ± 0.001 (< 10^{-16})
	Prediction accuracy	56.5% ± 0.1%	56.5% ± 0.1%	56.6% ± 0.1%
	PPV	56.2% ± 0.1%	56.2% ± 0.1%	55.9% ± 0.1%
	NPV	56.9% ± 0.2%	56.8% ± 0.2%	57.5% ± 0.2%
**Non-Specific Gastrointestinal Disorder** (50,791)	Nagelkerke R^2 (*p*-value)	–	0.022 ± 0.001 (2.2×10^{-7})	0.050 ± 0.001 (< 10^{-16})
	Prediction accuracy	–	53.3% ± 0.2%	53.2% ± 0.1%
	PPV	–	53.3% ± 0.2%	53.1% ± 0.1%
	NPV	–	53.4% ± 0.2%	53.4% ± 0.2%
**Pure Hypercholesterolemia** (41,826)	Nagelkerke R^2 (*p*-value)	–	0.167 ± 0.002 (< 10^{-16})	0.182 ± 0.001 (< 10^{-16})
	Prediction accuracy	–	63.9% ± 0.1%	64.1% ± 0.1%
	PPV	–	62.9% ± 0.1%	61.8% ± 0.1%
	NPV	–	65.1% ± 0.2%	67.5% ± 0.2%
**Upper Respiratory Inflammation** (35,974)	Nagelkerke R^2 (*p*-value)	–	0.032 ± 0.001 (2.4×10^{-14})	0.059 ± 0.001 (< 10^{-16})
	Prediction accuracy	–	54.7% ± 0.1%	54.8% ± 0.1%
	PPV	–	54.7% ± 0.1%	54.6% ± 0.1%
	NPV	–	54.8% ± 0.1%	55.1% ± 0.1%
**Non-Specific Urinary Disorder** (30,623)	Nagelkerke R^2 (*p*-value)	–	0.066 ± 0.001 (< 10^{-16})	0.090 ± 0.001 (< 10^{-16})
	Prediction accuracy	–	58.2% ± 0.1%	58.6% ± 0.1%
	PPV	–	57.9% ± 0.1%	59.2% ± 0.2%
	NPV	–	58.5% ± 0.1%	58.1% ± 0.1%
**Non-Specific Vascular Disease** (29,914)	Nagelkerke R^2 (*p*-value)	–	0.020 ± 0.001 (4.9×10^{-5})	0.050 ± 0.001 (< 10^{-16})
	Prediction accuracy	–	52.9% ± 0.1%	52.8% ± 0.1%
	PPV	–	52.9% ± 0.1%	52.7% ± 0.1%
	NPV	–	52.9% ± 0.1%	52.8% ± 0.1%
**Unspecified Lipid Metabolism Disorder** (29,282)	Nagelkerke R^2 (*p*-value)	–	0.178 ± 0.001 (< 10^{-16})	0.188 ± 0.001 (< 10^{-16})
	Prediction accuracy	–	64.6% ± 0.1%	64.6% ± 0.1%
	PPV	–	63.8% ± 0.1%	63.0% ± 0.1%
	NPV	–	65.6% ± 0.1%	66.7% ± 0.2%
	Nagelkerke R^2 (*p*-value)	–	0.019 ± 0.001 (7.3×10^{-6})	0.049 ± 0.001 (< 10^{-16})

**General Spondylosis Spine Disorder** (22,785)	Prediction accuracy	–	52.9% ± 0.1%	52.8% ± 0.1%
	PPV	–	52.9% ± 0.1%	52.7% ± 0.1%
	NPV	–	52.9% ± 0.1%	52.9% ± 0.1%
**Muscle Ligament Disorder** (21,500)	Nagelkerke R^2 (*p*-value)	–	0.024 ± 0.001 (2.6×10^{-8})	0.053 ± 0.001 ($< 10^{-16}$)
	Prediction accuracy	–	53.7% ± 0.1%	54.2% ± 0.2%
	PPV	–	53.6% ± 0.1%	54.1% ± 0.1%
	NPV	–	53.7% ± 0.1%	54.3% ± 0.2%
**Benign Colon Neoplasm** (19,909)	Nagelkerke R^2 (*p*-value)	–	0.084 ± 0.001 ($< 10^{-16}$)	0.106 ± 0.001 ($< 10^{-16}$)
	Prediction accuracy	–	59.3% ± 0.1%	59.5% ± 0.1%
	PPV	–	58.8% ± 0.1%	58.3% ± 0.1%
	NPV	–	59.9% ± 0.1%	61.2% ± 0.2%
**Non-Specific Skin Disorder** (19,667)	Nagelkerke R^2 (*p*-value)	–	0.018 ± 0.001 (9.8×10^{-5})	0.048 ± 0.001 ($< 10^{-16}$)
	Prediction accuracy	–	52.6% ± 0.1%	52.5% ± 0.1%
	PPV	–	52.6% ± 0.1%	52.5% ± 0.1%
	NPV	–	52.6% ± 0.1%	52.5% ± 0.1%

[a] We defined prediction accuracy as the number of correctly-classified samples to the total number of trials, based on the testing datasets;

[b] PPV: Positive predictive value; we defined it as the proportion of positive results (predicted as cases who had the disease of interest by polygenic prediction models) that are true positive, based on the testing datasets;

[c] NPV: Negative predictive value; we defined it as the proportion of negative results (predicted as controls who did not have the disease of interest by polygenic prediction models) that are true negative, based on the testing datasets.

Supplementary Table 3. Numbers of SNPs in associations with embedding dimensions.

As described in Methods section 6, we tested the associations between additive SNP effects and each of the 20 dimensions of individual-specific disease embedding coordinates. SNPs that met the Genome-wide significance ($p < 5\times10^{-8}$) are summarized in Supplementary Data 4. Here, we count the number of the identified SNPs in associations with each embedding dimension.

Embedding dimension	1	2	3	4	5	6	7	8	9	10	11	12	13	14	15	16	17	18	19	20
No. of identified SNPs	6	1	4	5	9	4	15	9	2	7	2	6	4	5	5	8	16	1	3	4

Supplementary Table 4. Summary counts of 547 diseases allocated to ten constellations.

Given the 20-dimensional embedding vectors of 547 diseases and of the identified ten disease constellations, we computed cosine similarity between a given disease and each of the ten disease constellations, and then claimed that the disease would belong to the disease constellation with which it had the largest cosine similarity value (see Supplementary Data 3 and Methods section 10.2). This table counts the 547 diseases' allocations in each of the ten constellations.

Disease constellation	1	2	3	4	5	6	7	8	9	10
Disease count	38	55	49	49	45	64	58	36	96	57

Supplementary Table 5. Summary counts of constellation assignments for UK Biobank's white British individuals.

Given diagnosis records, we can label the patient with all the possible disease constellations to which the diseases in her record belonged (see Methods section 10.2). The table below summarizes the constellation assignments for the 337,205 patients of white British background in the UK Biobank (please note that one patient can be assigned with multiple disease constellation labels).

Disease constellation	1	2	3	4	5	6	7	8	9	10
Patient count	118,639	116,839	136,017	85,727	66,509	199,408	47,036	24,346	60,348	176,029
