# Supplementary material for: The high-dimensional space of human diseases built from diagnosis records and mapped to genetic loci
Source: Nat Comput Sci. 2023 May 22;3(5):403–17. doi: 10.1038/s43588-023-00453-y (PMC10766526; doi:10.1038/s43588-023-00453-y)
Supplement: Supplementary file 2 — Reporting Summary [file 43588_2023_453_MOESM2_ESM.pdf]

nature portfolio

Corresponding author(s): Andrey Rzhetsky

Last updated by author(s): Apr 11, 2023

Reporting Summary

Nature Portfolio wishes to improve the reproducibility of the work that we publish. This form provides structure for consistency and transparency in reporting. For further information on Nature Portfolio policies, see our Editorial Policies and the Editorial Policy Checklist.

Statistics

For all statistical analyses, confirm that the following items are present in the figure legend, table legend, main text, or Methods section.

n/a	Confirmed	
☐	☒	The exact sample size (n) for each experimental group/condition, given as a discrete number and unit of measurement
☐	☒	A statement on whether measurements were taken from distinct samples or whether the same sample was measured repeatedly
☐	☒	The statistical test(s) used AND whether they are one- or two-sided<br>*Only common tests should be described solely by name; describe more complex techniques in the Methods section.*
☐	☒	A description of all covariates tested
☐	☒	A description of any assumptions or corrections, such as tests of normality and adjustment for multiple comparisons
☐	☒	A full description of the statistical parameters including central tendency (e.g. means) or other basic estimates (e.g. regression coefficient) AND variation (e.g. standard deviation) or associated estimates of uncertainty (e.g. confidence intervals)
☐	☒	For null hypothesis testing, the test statistic (e.g. F, t, r) with confidence intervals, effect sizes, degrees of freedom and P value noted<br>*Give P values as exact values whenever suitable.*
☐	☒	For Bayesian analysis, information on the choice of priors and Markov chain Monte Carlo settings
☒	☐	For hierarchical and complex designs, identification of the appropriate level for tests and full reporting of outcomes
☐	☒	Estimates of effect sizes (e.g. Cohen's d, Pearson's r), indicating how they were calculated

Our web collection on statistics for biologists contains articles on many of the points above.

Software and code

Policy information about availability of computer code

Data collection	No software was required for data collection (data preexisted and were provided by third parties).
Data analysis	We used R 4.1 and Python 3.8 to write our scripts. We provide a Code Ocean capsule including executable programming scripts, and input and output data at Jia, G. et al. The high-dimensional space of human diseases built from diagnosis records and mapped to genetic loci. Code Ocean https://doi.org/10.24433/CO.0096653.v1 (2023).

For manuscripts utilizing custom algorithms or software that are central to the research but not yet described in published literature, software must be made available to editors and reviewers. We strongly encourage code deposition in a community repository (e.g. GitHub). See the Nature Portfolio guidelines for submitting code & software for further information.

Data

Policy information about availability of data

All manuscripts must include a data availability statement. This statement should provide the following information, where applicable:

- Accession codes, unique identifiers, or web links for publicly available datasets
- A description of any restrictions on data availability
- For clinical datasets or third party data, please ensure that the statement adheres to our policy

Source data for Figs. 1-4 and Extended Data Figs. 1-2 are available with this manuscript. The license of MarketScan databases is available to purchase by Federal, nonprofit, academic, pharmaceutical, and other researchers. Access to the data is contingent on completing a data use agreement and purchasing the needed license. More information about licensing the MarketScan databases can be found at https://www.merative.com/documents/brief/Marketscan_explainer_general. The phenotypic and genetic datasets of UK Biobank used in this study are available via the UK Biobank data access process (see http://www.ukbiobank.ac.uk/register-apply/), and detailed information can be found at http://www.ukbiobank.ac.uk/scientists-3/genetic-data/ and http://biobank.ctsu.ox.ac.uk/crystal/label.cgi?id=100314. Access to the phenotypic and genetic datasets of BioVU can be requested after a study proposal is received, approved by the BioVU Review Committee and a user agreement is signed. More information can be found at https://victr.vumc.org/how-to-use-biovu/. The availability about the phenotypic and genetic datasets of Biobank Japan is described at https://biobankjp.org/english/index.html, and more information can be found at https://humandbs.biosciencedbc.jp/en/hum0014-v21.

Human research participants

Policy information about studies involving human research participants and Sex and Gender in Research.

Reporting on sex and gender	In all cohorts (MarketScan, BioVU, UK BioBank, BioBank Japan) we used the full gamut of populations characteristics, including sex and gender.
Population characteristics	We used sex, age, diagnostic data, clinical tests, and genotypic data when available.
Recruitment	Recruitment is not applicable because we used the existing de-identified data.
Ethics oversight	The research was approved by the University of Chicago Institutional Review Board, and passed ethical approval by the respective organizations that maintain the individual databases.

Note that full information on the approval of the study protocol must also be provided in the manuscript.

Field-specific reporting

Please select the one below that is the best fit for your research. If you are not sure, read the appropriate sections before making your selection.

☒ Life sciences ☐ Behavioural & social sciences ☐ Ecological, evolutionary & environmental sciences

For a reference copy of the document with all sections, see nature.com/documents/nr-reporting-summary-flat.pdf

Life sciences study design

All studies must disclose on these points even when the disclosure is negative.

Sample size	Sample size of each cohort was pre-determined by the data providers, therefore no special efforts were required in this regard.
Data exclusions	No data were excluded from the analyses.
Replication	Our replication strategies was to use UK Biobank as a discovery cohort, and BioBank Japan and BioVU as replication cohorts.
Randomization	Not applicable to our study design: randomization is applicable to clinical trials, but not required in observational studies.
Blinding	Not applicable to our study design: we did not assign treatment/placebo pairs as is done in clinical trials, but run observational association studies.

Reporting for specific materials, systems and methods

We require information from authors about some types of materials, experimental systems and methods used in many studies. Here, indicate whether each material, system or method listed is relevant to your study. If you are not sure if a list item applies to your research, read the appropriate section before selecting a response.

Materials & experimental systems

n/a	Involved in the study
☒	☐ Antibodies
☒	☐ Eukaryotic cell lines
☒	☐ Palaeontology and archaeology
☒	☐ Animals and other organisms
☒	☐ Clinical data
☒	☐ Dual use research of concern

Methods

n/a	Involved in the study
☒	☐ ChIP-seq
☒	☐ Flow cytometry
☒	☐ MRI-based neuroimaging
